# Comparative genomic analysis of strain *Priestia megaterium* B1 reveals conserved potential for adaptation to endophytism and plant growth promotion

**DOI:** 10.1128/spectrum.00422-24

**Published:** 2024-06-25

**Authors:** Fatma M. Mahmoud, Karin Pritsch, Roberto Siani, Sarah Benning, Viviane Radl, Susanne Kublik, Boyke Bunk, Cathrin Spröer, Michael Schloter

**Affiliations:** 1 Research Unit for Comparative Microbiome Analysis, Helmholtz Munich, German Research Center for Environmental Health, Neuherberg, Germany; 2 Botany and Microbiology Department, Faculty of Science, Suez Canal University, Ismailia, Egypt; 3 Research Unit for Environmental Simulations, Helmholtz Munich, German Research Center for Environmental Health, Neuherberg, Germany; 4 Leibniz Institute DSMZ-German Collection of Microorganisms and Cell Cultures GmbH, Braunschweig, Germany; 5 Chair for Environmental Microbiology, TUM School of Life Sciences, Technical University of Munich, Munich, Germany; Instituto de Ecología, A.C., Pátzcuaro, Michoacán, Mexico

**Keywords:** *Priestia *(*Bacillus*)* megaterium*, genome comparison, endophytes, plant-microbe interaction, pan genome, apple replant disease

## Abstract

In our study, we aimed to explore the genomic and phenotypic traits of *Priestia megaterium* strain B1, which was isolated from root material of healthy apple plants, to adapt to the endophytic lifestyle and promote plant growth. We identified putative genes encoding proteins involved in chemotaxis, flagella biosynthesis, biofilm formation, secretory systems, detoxification, transporters, and transcription regulation. Furthermore, B1 exhibited both swarming and swimming motilities, along with biofilm formation. Both genomic and physiological analyses revealed the potential of B1 to promote plant growth through the production of indole-3-acetic acid and siderophores, as well as the solubilization of phosphate and zinc. To deduce potential genomic features associated with endophytism across members of *P. megaterium* strains, we conducted a comparative genomic analysis involving 27 and 31 genomes of strains recovered from plant and soil habitats, respectively, in addition to our strain B1. Our results indicated a closed pan genome and comparable genome size of strains from both habitats, suggesting a facultative host association and adaptive lifestyle to both habitats. Additionally, we performed a sparse Partial Least Squares Discriminant Analysis to infer the most discriminative functional features of the two habitats based on Pfam annotation. Despite the distinctive clustering of both groups, functional enrichment analysis revealed no significant enrichment of any Pfam domain in both habitats. Furthermore, when assessing genetic elements related to adaptation to endophytism in each individual strain, we observed their widespread presence among strains from both habitats. Moreover, all members displayed potential genetic elements for promoting plant growth.

**IMPORTANCE:**

Both genomic and phenotypic analyses yielded valuable insights into the capacity of *P. megaterium* B1 to adapt to the plant niche and enhance its growth. The comparative genomic analysis revealed that *P. megaterium* members, whether derived from soil or plant sources, possess the essential genetic machinery for interacting with plants and enhancing their growth. The conservation of these traits across various strains of this species extends its potential application as a bio-stimulant in diverse environments. This significance also applies to strain B1, particularly regarding its application to enhance the growth of plants facing apple replant disease conditions.

## INTRODUCTION

Plant colonization by endophytes encompasses a sequence of events, starting with encountering root exudates (chemotaxis), the movement toward the roots, adherence to the root surface, formation of biofilm, root penetration, and, ultimately, proliferation in the root tissues (1). Additionally, bacterial secretion systems and overcoming plant immune reactions play pivotal roles in the initial stages of plant-microbe interaction (2). As revealed by comprehensive analysis of entire genomes of endophytes, the crucial genes associated with endophytism incorporate genes encoding proteins involved in chemotaxis, motility, secretion, adhesion, and biofilm formation (2). This was also emphasized by studies that demonstrated that mutants deficient in such genes exhibited decreased capacity for root colonization, as reviewed by Pinski et al. (1). Additionally, genome mining of plant growth-promoting endophytes revealed genes related to solubilization of phosphate, production of siderophores which promote nutrient acquisition, and biosynthesis of indole-3-acetic acid (3–5).

Comparative genomics of endophytic and non-endophytic isolates unveiled characteristics involved in establishing endophytic behavior. In a study conducted by Hardoim et al. (6), genomes of 40 endophytic bacterial strains were compared with 42 nodule symbionts, 29 phytopathogens, 42 rhizosphere strains, and 49 soil ones. Their results indicated a higher abundance of genes encoding proteins related to chemotaxis and motility (e.g., Tar, Tap, CheBR, and CheC) in endophytes compared to the other groups. Moreover, genes related to signal transduction, transcriptional regulators, and detoxification were more pronounced in endophytes (6). Levy et al. (7) compared 3,837 genomes representing various bacterial taxa from different isolation origins and classified these into three main categories: plant, soil, and non-plant associated. Their results showed that plant-associated bacteria were enriched in genetic elements involved in carbohydrate metabolism and depleted in mobile elements in comparison to non-plant-associated genomes (7). Bünger et al. (8) demonstrated the enrichment of 19 Pfam domains related to flagellar motility in endophytes, compared to soil strains (8). Also, leaf-associated strains exhibited significant enrichment in genes responsible for adaptation to the environment (e.g., cytochrome P450 and chemotaxis), while genes related to transcription regulation and sporulation were more abundant in soil-associated strains (9).

*Priestia megaterium* [previously known as *Bacillus megaterium* (10)] is a Gram-positive, rod-shaped, spore-forming bacterium (11) which has been known for its antimicrobial activity against different phytopathogens (12–14). Several strains have been found to exhibit diverse plant growth-promoting characteristics, such as the solubilization of zinc (15) and phosphorus (16), as well as the production of siderophores and indole-3-acetic acid (17). *P. megaterium* has been isolated from diverse habitats, including soil (18, 19) and plant tissues (20, 21). However, it remains unclear whether the differentiation between soil and endophytic strains arises from strain-specific differences or if such bacteria carry traits important for survival in soil as well as colonization of roots.

We isolated stain *P. megaterium* B1 from healthy roots of apple plantlets (22), with a future aim to improve growth of apple seedlings mainly in soils which are affected by apple replant disease. In this study, we focused on investigating the genomic and phenotypic traits of B1 related to adaptation to the plant niche and enhancing plant growth. Additionally, we conducted a comparative genomic analysis of strain B1 with other *P. megaterium* strains derived from plants and soil to identify potential genetic markers differentiating plant- and soil-derived strains and to enhance our understanding of genetic elements that may contribute to plant association of strain B1.

## RESULTS

### General genomic features of *P. megaterium* B1

The PacBio sequencing run resulted in 812,984 reads (mean read length: 4,361.93 bp; N50: 4,553 bp), while Illumina MiSeq sequencing resulted in 22,081,279 paired-end reads (read length 301 bp). Filtering and trimming of Illumina reads resulted in a total number of 21,750,560 high-quality paired-end reads (mean read length: 200 bp), which were used for polishing the *de novo* assembled genome. The polished *de novo* assembled genome (accession number GCA_024582855.4) was ≈5.4 Mb in length, with scaffold N50: ≈5.1 Mb and a GC content of 38.05%. It comprised five contigs (one chromosomal contig, one megaplasmid, and three circular plasmids). The high-quality genome displayed a completeness of 99.4% and a contamination of 0.07%.

**Fig 1:**
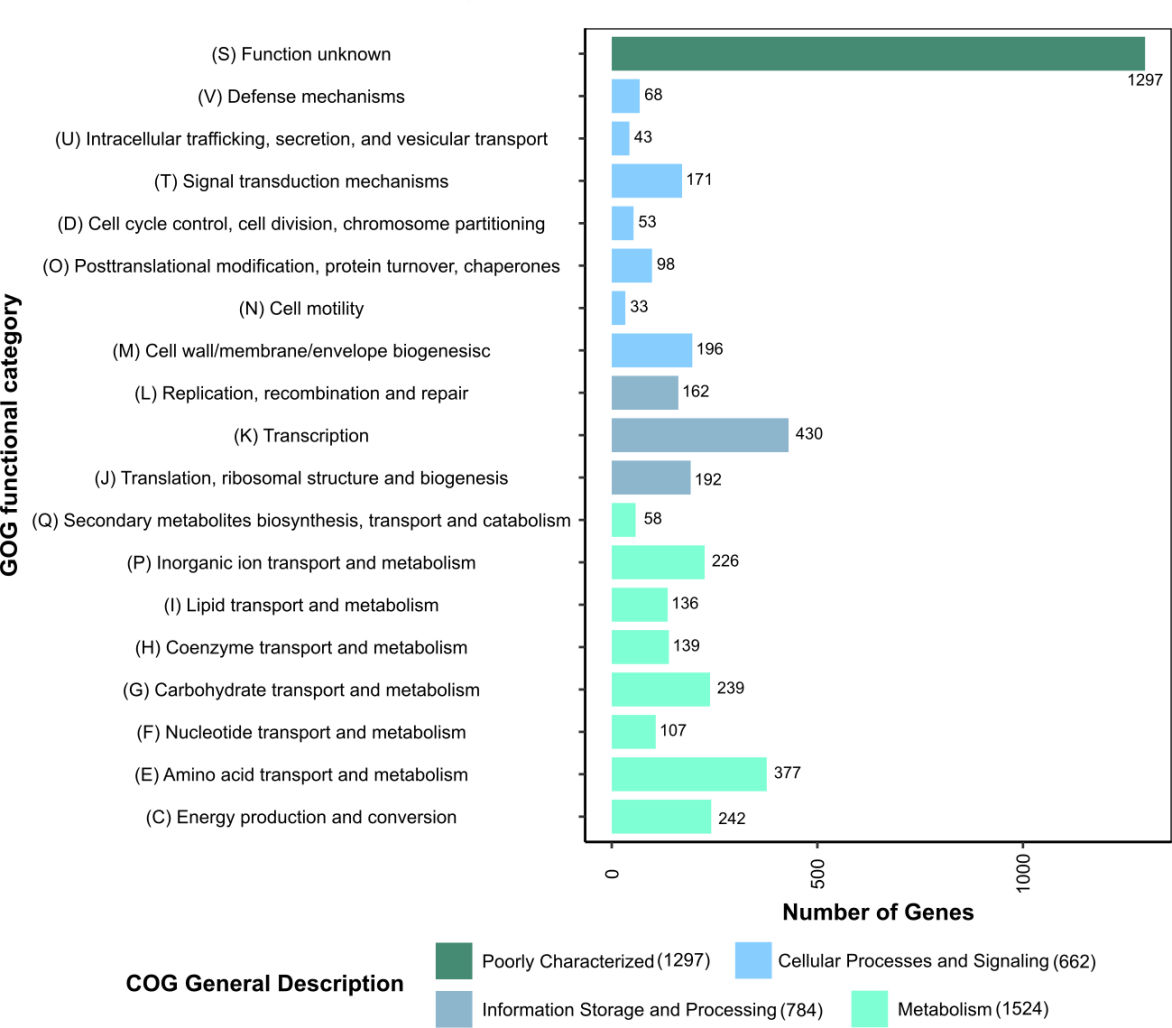
COG functional characterization of *P. megaterium* B1 coding DNA sequences. The numbers presented on the bars and beside the legend levels state the number of genes that belong to each category.

Prokka identified 5,506 coding DNA sequences (CDSs), in addition to 42 rRNA, 125 tRNA, and 1 tmRNA coding genes. EggNOG-mapper assigned 4,267 CDSs to different COGs (Cluster of Orthologous Groups of proteins) classes, where 69.6% were assigned to known functions. The majority of these genes were predicted to be involved in primary and secondary metabolisms (Fig. 1).

### Potential of *P. megaterium* B1 for adapting to plant environment

The analysis of the annotated genome of B1 revealed genes that might contribute to its interaction with plants and adaptation to the plant niche (Table 1). The genome of B1 harbored genes encoding the chemotaxis proteins MCPs, CheA, CheW, CheY, CheR, CheB, and CheD. Genes involved in biosynthesis of flagella and motility were also detected in the genome of B1. Genes encoding flagellin FliC, hook protein FlgE, and hook length control protein FliK were also recognized. Moreover, B1 possessed genes coding type III export proteins FlhA, FlhB, Flip, FliQ, FliR, FliH, FliI, and FliJ. M-ring and C-ring protein encoding genes (*fliF* and *fliG*, *fliM*, *fliN*, *fliY*), respectively, were also detected in the genome of B1. Finally, stator protein biosynthesis genes *motA* and *motB* were identified. The *lapA* gene, encoding lipopolysaccharide A, which plays a role in biofilm formation, was also detected. The potential motility of B1 was confirmed, as it exhibited both swarming and swimming motilities (Fig. S1A and B). Additionally, it displayed the ability for biofilm formation (Fig. S2). The genome of B1 also revealed genetic elements related to different secretory systems, including Sec translocase (*secA*, *secD*, *secyY*, *secE*, and *secG*), twin-arginine translocase (Tat) (*tatA* and *tatC*), sortase (*strD*), as well as components of type VII secretion system (*esaB*, *esaA*, *essB*, and *essC*). Genes *katE* and *sodA* encoding catalase and superoxide dismutase, respectively, were identified in the genome of B1. The genome of B1 also revealed a total of 81 genes predicted by all dbCAN databases that encode carbohydrate-active enzymes (CAZymes), including glycoside hydrolases (23), glycosyltransferases (23), carbohydrate esterases (14) carbohydrate-binding molecules (4), and polysaccharide lyases (1) (Table S1). Genes belonging to glycoside hydrolase (GH) families 36, 28, and 1, which encompass enzymes involved in breakdown of hemicellulose, pectin, and cellulose, were identified. Additionally, α-amylase (GH13) and α-glucosidase (GH31) encoding genes, which are included in metabolism of starch, were also recognized.

**TABLE 1:** Genetic elements involved in interaction with plants

Locus-tag	Gene	KEGG/COG/Pfam^*a*^	Product	Pathway
DKPENOPO_03987	*cheA*	K03407	Two-component system, chemotaxis family, sensor kinase CheA	Chemotaxis proteins
DKPENOPO_03986	*cheW*	K03408	Purine-binding chemotaxis protein CheW	
DKPENOPO_03985	*cheD*	K03411	Chemotaxis protein CheD	
DKPENOPO_04143	*cheR*	K00575	Chemotaxis protein methyltransferase CheR	
DKPENOPO_01377				
DKPENOPO_03988	*cheB*	K03412	Two-component system, chemotaxis family, protein-glutamate methylesterase/glutaminase	
DKPENOPO_02086	*cheBR*	K13924	Two-component system, chemotaxis family, CheB/CheR fusion protein	
DKPENOPO_03997	*cheY*	K03413	Two-component system, chemotaxis family, chemotaxis protein CheY	
DKPENOPO_01088	*cheV*	K03415	Two-component system, chemotaxis family, chemotaxis protein CheV	
DKPENOPO_00298	*mcp*	K03406	Methyl-accepting chemotaxis protein	
DKPENOPO_00896				
DKPENOPO_00972				
DKPENOPO_02280				
DKPENOPO_03337				
DKPENOPO_03711	*hemAT*	K06595	Heam-based aerotactic transducer	
DKPENOPO_04007	*fliH*	K02411	Flagellar assembly protein FliH	Flagellar proteins
DKPENOPO_04006	*fliI*	K02412	Flagellum-specific ATP synthase	
DKPENOPO_04005	*fliJ*	K02413	Flagellar protein FliJ	
DKPENOPO_03996	*fliO*/*fliZ*	K02418	Flagellar protein FliO/FliZ	
DKPENOPO_03995	*fliP*	K02419	Flagellar biosynthesis protein FliP	
DKPENOPO_03994	*fliQ*	K02420	Flagellar biosynthesis protein FliQ	
DKPENOPO_03993	*fliR*	K02421	Flagellar biosynthesis protein FliR	
DKPENOPO_03991	*flhA*	K02400	Flagellar biosynthesis protein FlhA	
DKPENOPO_03992	*flhB*	K02401	Flagellar biosynthesis protein FlhB	
DKPENOPO_04008	*fliG*	K02410	Flagellar motor switch protein FliG	
DKPENOPO_03999	*fliM*	K02416	Flagellar motor switch protein FliM	
DKPENOPO_03998	*fliN*	K02417	Flagellar motor switch protein FliN	
DKPENOPO_03998	*fliY*	PF04509	Flagellar motor switch phosphatase FliY/CheC-like protein	
DKPENOPO_04009	*fliF*	K02409	Flagellar M-ring protein FliF	
DKPENOPO_04000	*fliL*	K02415	Flagellar protein FliL	
DKPENOPO_04010	*fliE*	K02408	Flagellar hook-basal body complex protein FliE	
DKPENOPO_04003	*fliK*	K02414	Flagellar hook-length control protein FliK	
DKPENOPO_04012	*flgB*	K02387	Flagellar basal-body rod protein FlgB	
DKPENOPO_04011	*flgC*	K02388	Flagellar basal-body rod protein FlgC	
DKPENOPO_04002	*flgD*	K02389	Flagellar basal-body rod modification protein FlgD	
DKPENOPO_04961	*flgF*	K02391	Flagellar basal-body rod protein FlgF	
DKPENOPO_04960	*flgG*	K02392	Flagellar basal-body rod protein FlgG	
DKPENOPO_04001	*flgE*	K02390	Flagellar hook protein FlgE	
DKPENOPO_04933	*flgK*	K02396	Flagellar hook-associated protein 1	
DKPENOPO_04932	*flgL*	K02397	Flagellar hook-associated protein 3 FlgL	
DKPENOPO_00876	*fliC*/*hag*	K02406	Flagellin	
DKPENOPO_04923	*fliD*	K02407	Flagellar hook-associated protein 2	
DKPENOPO_04922	*fliS*	K02422	Flagellar secretion chaperone FliS	
DKPENOPO_04941				
DKPENOPO_04921	*fliT*	K02423	Flagellar protein FliT	
DKPENOPO_03984	*fliA*	K02405	RNA polymerase sigma factor FliA	
DKPENOPO_01793	*motA*	K02556	Chemotaxis protein MotA	
DKPENOPO_04631				
DKPENOPO_01794	*motB*	K02557	Chemotaxis protein MotB	
DKPENOPO_04630				
DKPENOPO_04931	*fliW*	K13626	Flagellar assembly factor FliW	
DKPENOPO_04935	*flgM*	K02398	Negative regulator of flagellin synthesis FlgM	
DKPENOPO_03990	*flhF*	K02404	Flagellar biosynthesis protein FlhF	
DKPENOPO_03989	*flhG*	K04562	Flagellar biosynthesis protein FlhG	
DKPENOPO_00115	*esaB*	COG5417	EsaB/YukD family protein	Secretory proteins
DKPENOPO_00118	*esaA*	COG1511	Type VII secretion protein EsaA	
DKPENOPO_00116	*essB*	COG4499	Type VII secretion system protein EssB	
DKPENOPO_00117	*essC*	COG1674	Type VII secretion protein EssC	
DKPENOPO_04914	*tatC*		Twin-arginine translocase subunit TatC	
DKPENOPO_05362				
DKPENOPO_00771				
DKPENOPO_04915	*tatA*		Twin-arginine translocase TatA/TatE family subunit	
DKPENOPO_01670				
DKPENOPO_05361				
DKPENOPO_04449	*secDF*	COG0341	Protein translocase subunit SecDF	
DKPENOPO_04866	*secG*	COG1314	Putative protein-export membrane protein SecG	
DKPENOPO_04918	*secA*	COG0653	Protein translocase subunit SecA	
DKPENOPO_05221	*secE*		Protein translocase subunit SecE	
DKPENOPO_05256	*secY*		Protein translocase subunit SecY	
DKPENOPO_03002	*srtD*		Sortase D	Secretion
DKPENOPO_03457				
DKPENOPO_00968	*lapA*		Lipopolysaccharide assembly protein A	Biofilm
DKPENOPO_02867	*katA*	COG0753	Catalase	Stress protection and detoxification
DKPENOPO_05062				
DKPENOPO_04330	*sodA*		Superoxide dismutase	
DKPENOPO_04767				
DKPENOPO_03900	*yheH*/ *yheI*		Putative multidrug resistance ABC transporter ATP-binding/permease protein YheH and YheI	
DKPENOPO_04406	*sigK*		RNA polymerase sigma-28 factor	Transcriptional regulators
DKPENOPO_04417	*greA*		Transcription elongation factor GreA	

^*a*^ KEGG, Kyoto Encyclopedia of Genes and Genomes.

### Potential of *P. megaterium* B1 for plant growth promotion

In addition to traits which determine plant-microbe interactions, B1 harbored genes related to plant growth promotion (Table 2). Genes involved in the biosynthesis of indole-3-acetic acid via the indole-3-pyruvic acid pathway were detected. This included *trpA* and *trpB* genes, which encode tryptophan biosynthesis. A putative aminotransferase encoding gene, which catalyzes the conversion of tryptophan to indole-3-pyruvate, was also detected. Additionally, the *padC* gene, which is involved in the transformation of indole-3-pyruvate to indole-3-acetaldehyde, was identified, as well as the putative aldehyde dehydrogenase gene, which is responsible for the conversion of indole-3-acetaldehyde to indole-3-acetic acid (IAA). Genome mining using antiSMASH showed that B1 possesses the gene cluster of biosynthesis of siderophores (Table 3). Additionally, genome annotation revealed potential genes involved in siderophore transport, including *yusV*, *yfhA*, *yfiZ*, *yfhA*, and *yfiY* genes (Table 2). The potential of B1 to solubilize phosphate was highlighted by the presence of genes encoding alkaline phosphatases (*phoD*, *phoA*, and *phoB*). Besides, genes involved in the biosynthesis and transport of the two organic acids malate and citrate were identified in the genome of B1. Genes coding for phosphate transporters (*pstS*, *pstC*, and *pstB*) were also detected (Table 2). B1 possesses putative genetic elements involved in different mechanisms of solubilization of zinc, including organic acids, and production of chelating agents (e.g., siderophores) (Tables 2 and 3). Genetic plant growth promotion potential of B1 was further confirmed by physiological tests. B1 produced indole-3-acetic acid (Fig. S3A) in the concentration of 5.23 µg/mL. Additionally, it was able to solubilize calcium phosphate, incorporated in Pikovskayas (PVK) agar medium (Fig. S3B), and the phosphate solubilization index (SI) was estimated as 1.14 ± 0.05. B1 also tested positive for solubilization of zinc (Fig. S3C) with a zinc SI of 1.48 ± 0.1, in addition to production of siderophores (Fig. S3D).

**TABLE 2:** Genetic elements involved in plant growth promotion

Locus-tag	Gene	COG	Product	Pathway
DKPENOPO_04135	*trpA*	COG0159	Tryptophan synthase alpha chain	L-tryptophan production
DKPENOPO_04136	*trpB*	COG0133	Tryptophan synthase beta chain	
DKPENOPO_03859		COG0161	putative aminotransferase	Tryptophan conversion to indole-3-pyruvic acid
DKPENOPO_00146	*padC*	COG3479	Phenolic acid decarboxylase	Indole-3-pyruvate transformation to indole-3-acetaldehyde
DKPENOPO_01395		COG1012	Putative aldehyde dehydrogenase	indole-3-acetaldehyde conversion to Indole-3-acetic acid (IAA)
DKPENOPO_04828	*yusV*	COG1120	Putative siderophore transport system ATP-binding protein	Siderophores transport
DKPENOPO_01298				
DKPENOPO_04829	*yfhA*	COG0609	putative siderophore transport system permease protein YfhA	
DKPENOPO_01295				
DKPENOPO_00657				
DKPENOPO_04830	*yfiZ*	COG0609	putative siderophore transport system permease protein	
DKPENOPO_00656				
DKPENOPO_04830				
DKPENOPO_04831	*yfiY*	COG0614	putative siderophore-binding lipoprotein YfiY	
DKPENOPO_04916	*phoD*	COG3540	Alkaline phosphatase D	Solubilization of organic phosphate
DKPENOPO_01284	*phoA*	COG1785	Alkaline phosphatase 4	
DKPENOPO_00995	*phoB*	COG1785	Alkaline phosphatase 3	
DKPENOPO_01940	*phoP*	COG0745	Alkaline phosphatase synthesis transcriptional regulatory protein	
DKPENOPO_02304				
DKPENOPO_02753				
DKPENOPO_04580				
DKPENOPO_02753				
DKPENOPO_04579	*phoR*	COG0642	Alkaline phosphatase synthesis sensor protein	
DKPENOPO_04584	*citZ*	COG0372	Citrate synthase 2	Solubilization of inorganic phosphate
DKPENOPO_02911	*citA*	COG0372	Citrate synthase 1	
DKPENOPO_01811	*cimH*	COG3493	Citrate/malate transporter	
DKPENOPO_02722	*glcB*	COG2225	Malate synthase G	
DKPENOPO_05066	*yflS*	COG0471	Putative malate transporter YflS	
DKPENOPO_01799	*mleN*	COG1757	Malate-2H(+)/Na(+)-lactate antiporter	
DKPENOPO_04326	*phoU*	COG0704	Phosphate-specific transport system accessory protein	Phosphate transport
DKPENOPO_04438	*pstS*	COG0226	Phosphate-binding protein	
DKPENOPO_04437	*pstC*	COG0573	Phosphate transport system permease protein	
DKPENOPO_04327	*pstB*	COG1117	Phosphate import ATP-binding protein	

**TABLE 3:** Predicted biosynthetic gene clusters using antiSMASH v.7.0.1

Type	From	To	Most similar known cluster	Similarity (%)
NI siderophores	3,677,021	3,711,596	Synechobactin C9/synechobactin C11/synechobactin 13/synechobactin 14/synechobactin 16/synechobactin A/synechobactin B/synechobactin C	23
Terpene	3,505,098	3,525,916	Surfactin	13
Terpene	1,899,536	1,921,404	–	–
T3PKS	1,198,424	1,239,509	–	–
Phosphonate	523,147	540,568	–	–
Terpene	378,922	399,770	Carotenoid	50

Prediction of biosynthetic gene clusters, using antiSMASH, revealed surfactins encoding cluster with 13% similarity to best-matching known clusters. Other biosynthetic gene clusters were also predicted, including these encoding carotenoid and phosphonates. A biosynthetic gene cluster encoding unknown type III polyketide synthase, was also identified (Table 3).

### Pan-genome and phylogenetic analyses

**Fig 2:**
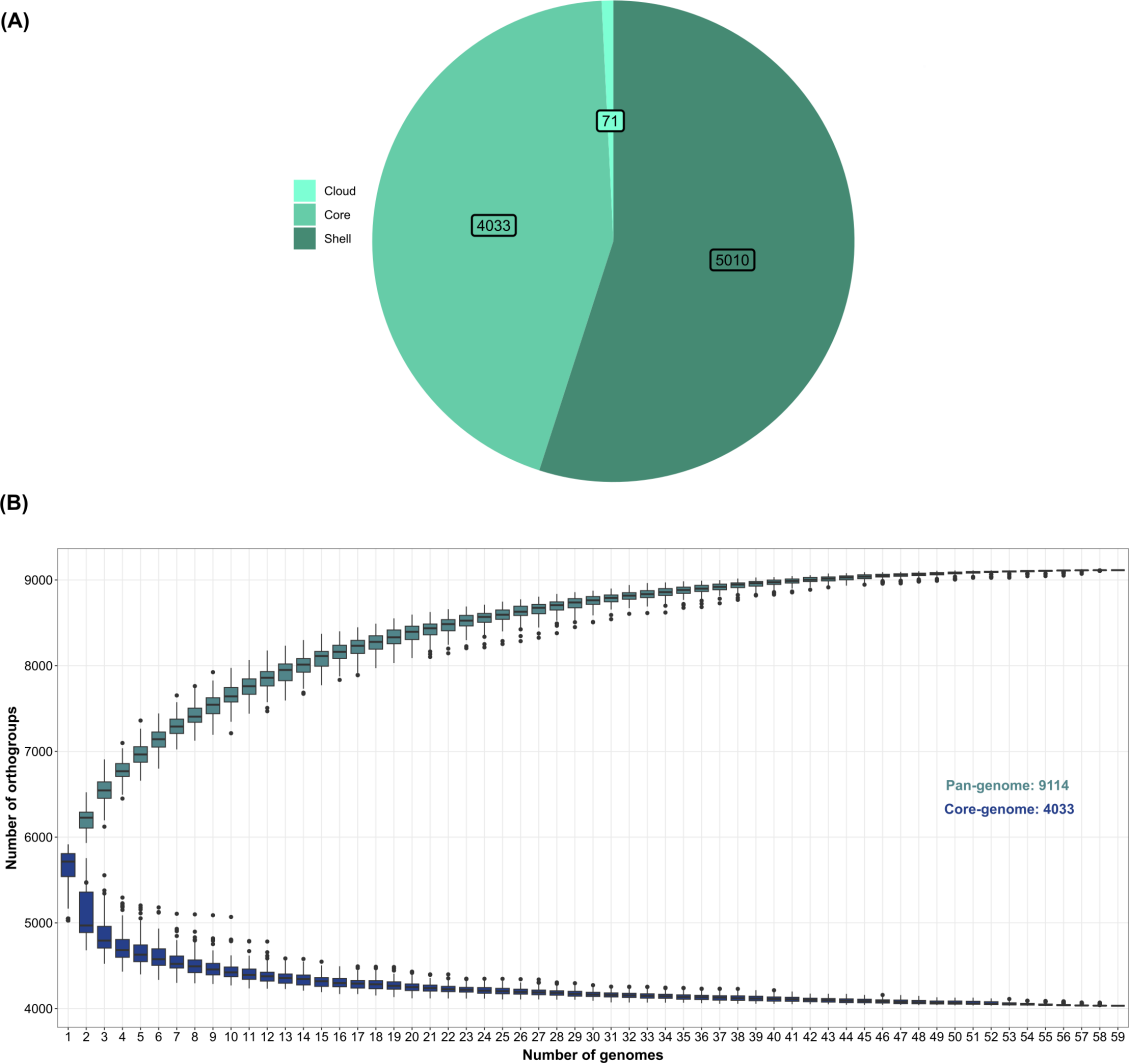
Pan-genome analysis of 59 strains. Graphs are based on the orthogroup gene count output generated by OrthoFinder v.2.5.5, which was subsequently transformed into a presence-absence matrix. (**A**) Pan-genome statistics. The numbers represent the number of orthogroups belonging to core genome (shared by all strains), shell genome (shared by the majority of strains but not all), and cloud genome (present in single strains). (**B**) Cumulative curves illustrate the number of orthologous protein clusters (orthogroups) of the pan and core genomes of plant and soil *P. megaterium* in relation to the number of genomes.

The pan-genome analysis based on the annotated protein sequences of 59 strains resulted in 346,252 genes assigned to 9,114 orthogroups representing the pan genome. A total of 4,033 orthogroups (44.25%) were conserved in all of 59 strains, among which 3,486 orthogroups were single copy. Also, 5,010 orthogroups (54.97%) represented the shell genome, while 71 orthogroups (0.78%) represented the cloud (strain-specific) genome (Fig. 2A). The α value was estimated as 1.07, indicating a closed pan genome of selected *P. megaterium* strains. This was also shown by the cumulative curve of the pan genome, as by adding more genomes, the number of orthogroups in the pan genome tended to stabilize. Additionally, the cumulative curve of the core genome indicated a declining trend of the number of core orthogroups as more genomes are included (Fig. 2B).

**Fig 3:**
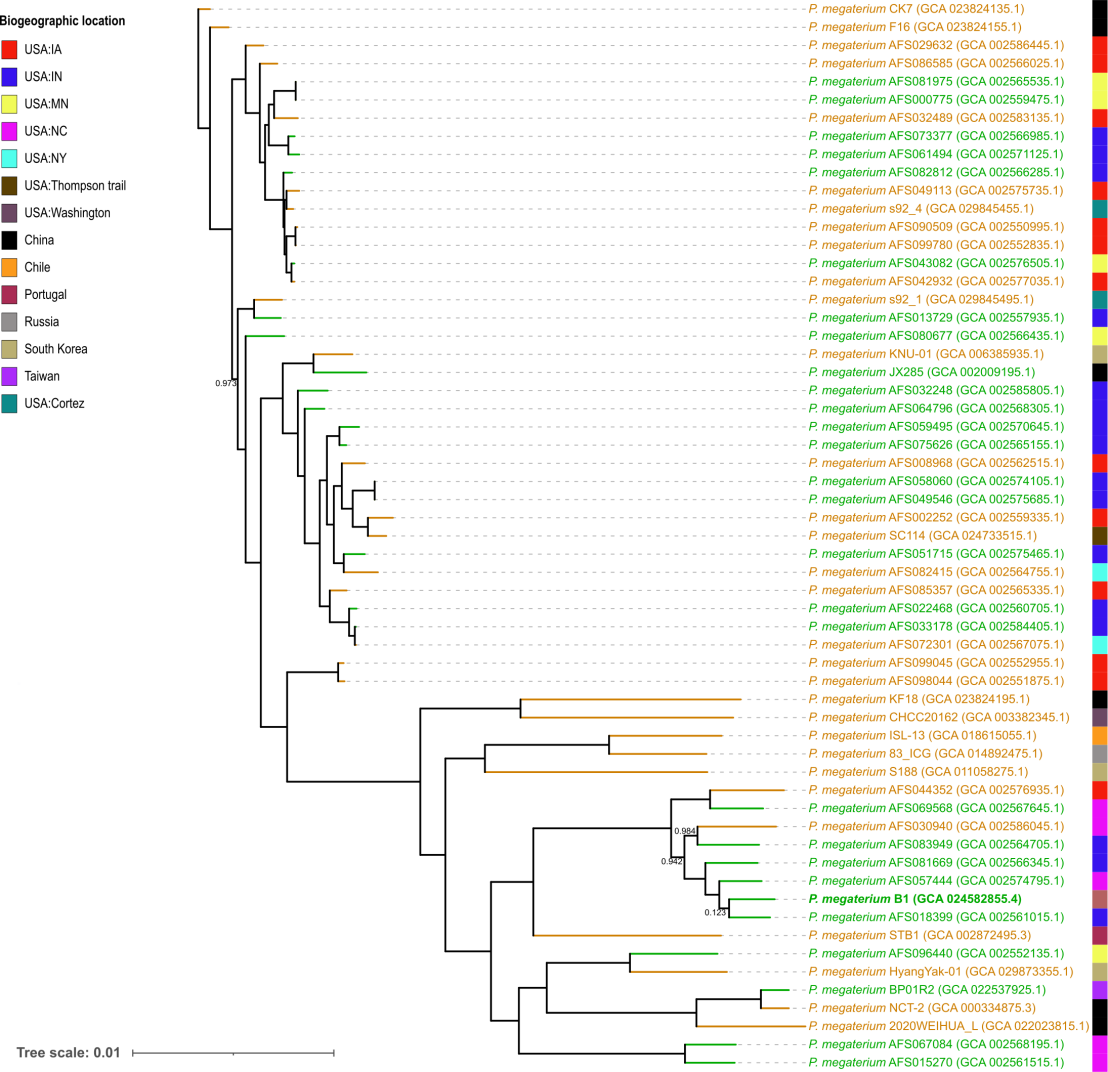
A maximum-likelihood phylogenetic tree constructed using OrthoFinder v.2.5.5, based on a concatenated multiple sequencing alignment (MSA) of amino acid sequences of 3,486 single-copy core orthogroups of 59 strains. The tree was inferred applying FastTree, where the support values reported on the branches refer to the bootstrap replicates derived from the full concatenated multigene MSA. Only support values of <1 are shown. The scale bar indicates the number of amino acid substitutions per site. Green color denotes plant strains; while brown color denotes soil strains. National Center for Biotechnology Information (NCBI) genome accession numbers are indicated between parentheses. The colored blocks beside the tip labels indicate the biogeographical location, stated by NCBI metadata.

The phylogenetic tree based on multiple sequence alignment of protein sequences of single-copy core orthogroups of 59 strains showed that the strains of soil and plant environments did not cluster in a distinctive pattern according to their different habitats or biogeographical location (Fig. 3). However, strain B1 clustered in the same clade with other strains of plant origin (GCA_002574795, GCA_002561015, and GCA_002566345) and displayed the highest average nucleotide identity (ANI) percentage with the three strains (Fig. S4).

**Fig 4:**
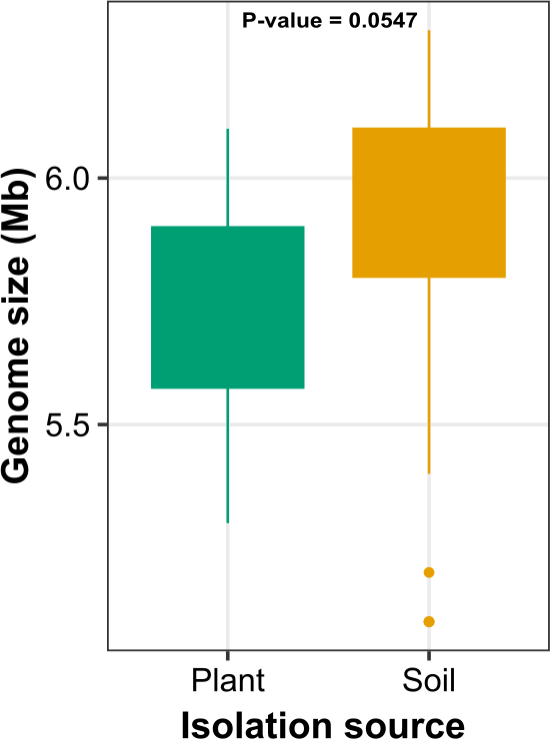
Boxplot showing the genome size of strains recovered from plant and soil habitats. The *P* value is estimated by Wilcoxon test implemented in R package rstatix v.0.7.2.

The genome size of strains which originated from plants and soil did not differ significantly (Fig. 4). The size of plant-derived genomes ranged from 5.3 to 6.1 Mb, while genomes of soil strains displayed a range of 5.1–6.3.

### Functional comparative genomic analysis

**Fig 5:**
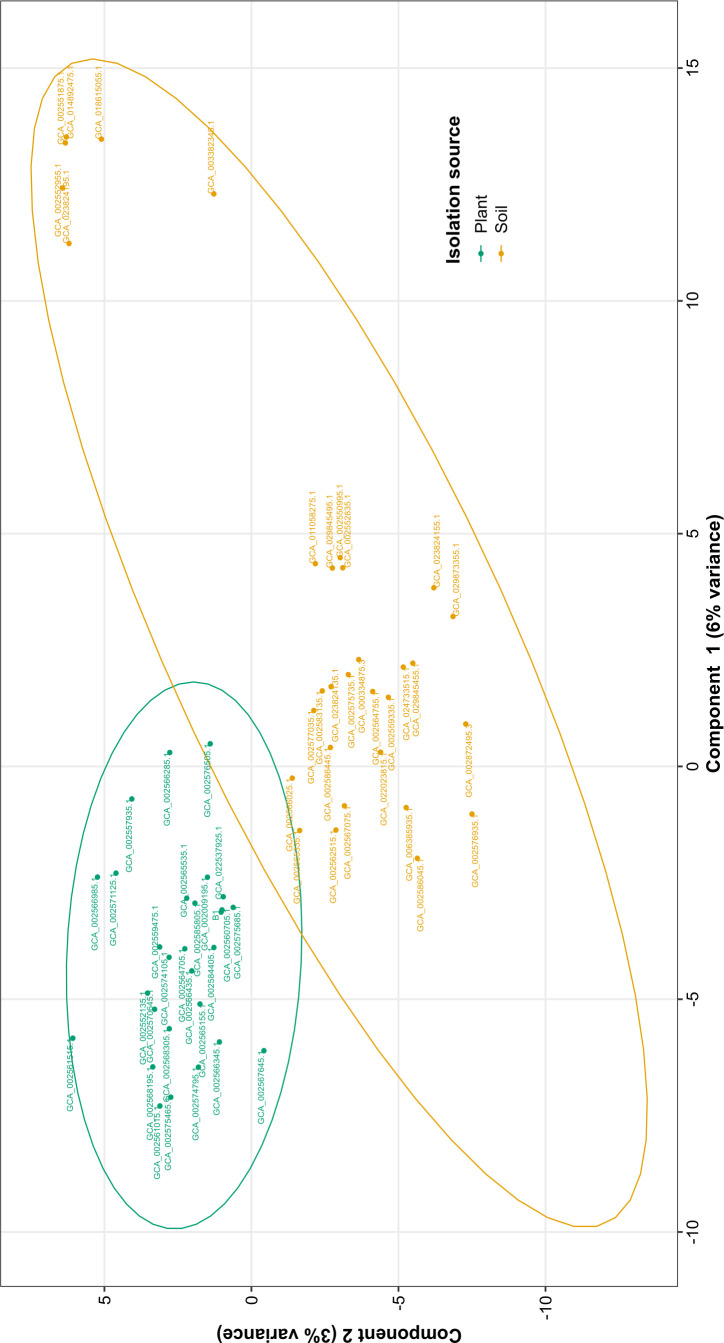
sPLS-DA sample plot showing distinctive clustering of plant and soil strains. The plot was generated using a presence-absence matrix of Pfam domains from 59 strains of *P. megaterium,* using R package “mixOmics v.6.24.0. Strains recovered from plant and soil habitats are represented by green and brown colors, respectively.

**Fig 6:**
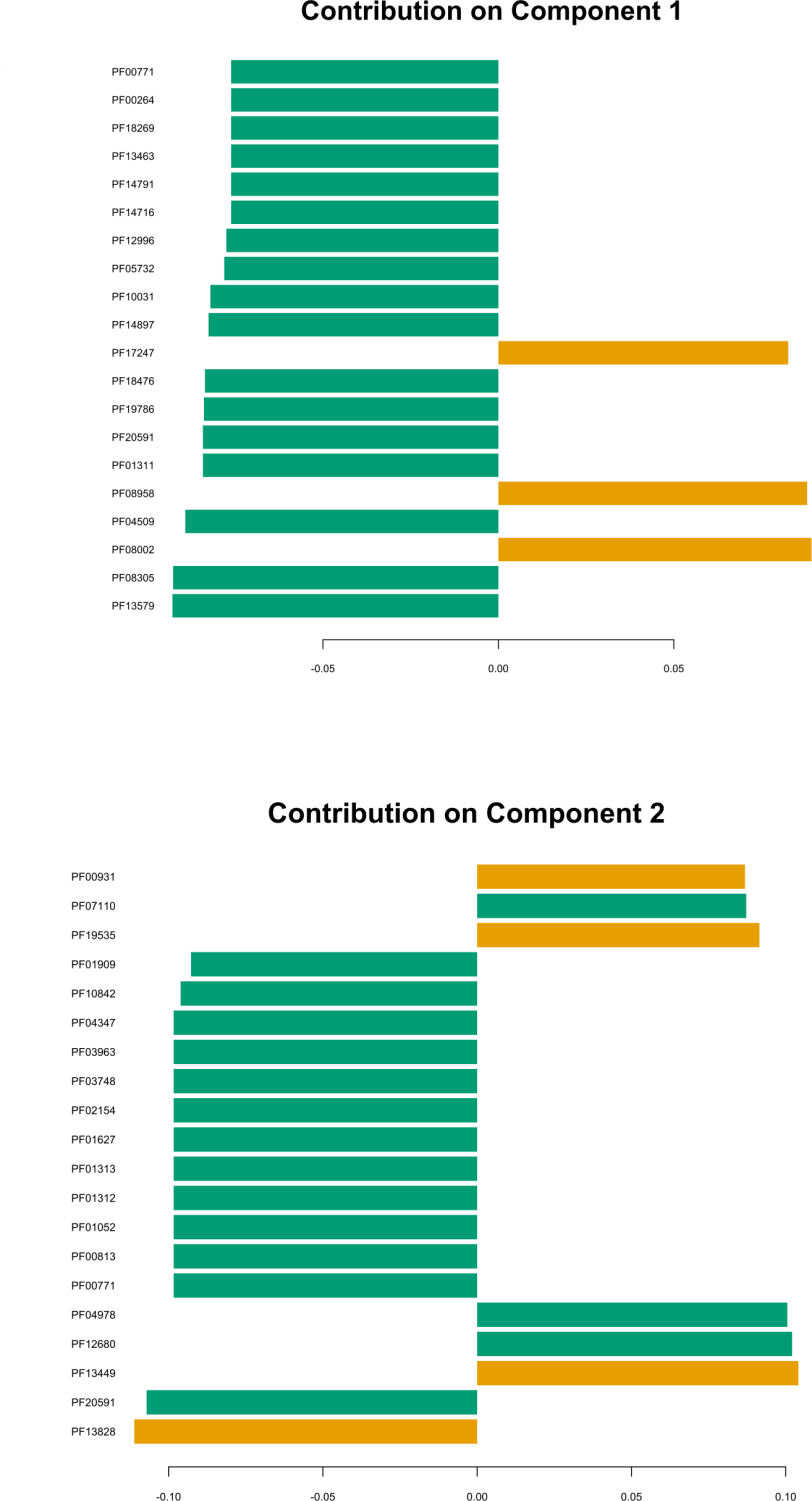
Barplot illustrates the loading weights of the top 20 Pfam domains contributing to components 1 and 2. Pfam domains are arranged in descending order according to their loading weight, with the most important at the bottom and the least important at the top. Greater absolute values in the loading vector indicate higher "importance" for a given Pfam domain. The color of each bar corresponds to the group with the higher mean of the selected Pfam domain. This indicates a greater representation of the domain in that group compared to the other. Plant and soil groups are represented by green and brown, respectively. The barplot was generated using the "plotLoadings()" function from the mixOmics package v.6.24.0, based on the sPLS-DA model.

Genomic comparison could reveal characteristic features associated with specific habitats. We conducted a functional comparative genomic analysis to identify functional traits that could be possibly associated with *P. megaterium* strains originating from plants (including *P. megaterium* B1), contrasting them with strains from soil environment. To investigate the discrimination between plant and soil strains and to which group B1 would relate more, sparse Partial Least Squares Discriminant Analysis (sPLS-DA) was performed using a matrix representing presence-absence of different Pfam domains in each strain. Strains derived from plants displayed a distinctive clustering from soil strains (Fig. 5), where components 1 and 2 accounted for 6% and 3% of the variance, respectively. Pfam domains PF04509, PF01052, PF02154, PF03748, PF03963, and PF04347, associated with motility and flagella biosynthesis (Table S2A and B), were among top 20 contributors to such clustering, where they showed higher representation in plant habitat (Fig. 6). However, functional enrichment analysis of Pfam domains, including these domains, showed no significant difference between the two habitats (false discovery rate (FDR) = 1) (Table S3). Moreover, in our data set, we identified 87 and 91 Pfam domains that were found by Levy et al. (7) to be significantly associated with plant/root and soil strains of *Bacillales*, respectively. However, we did not observe significant enrichment of any of these domains in the two habitats (Table S4A and B).

**Fig 7:**
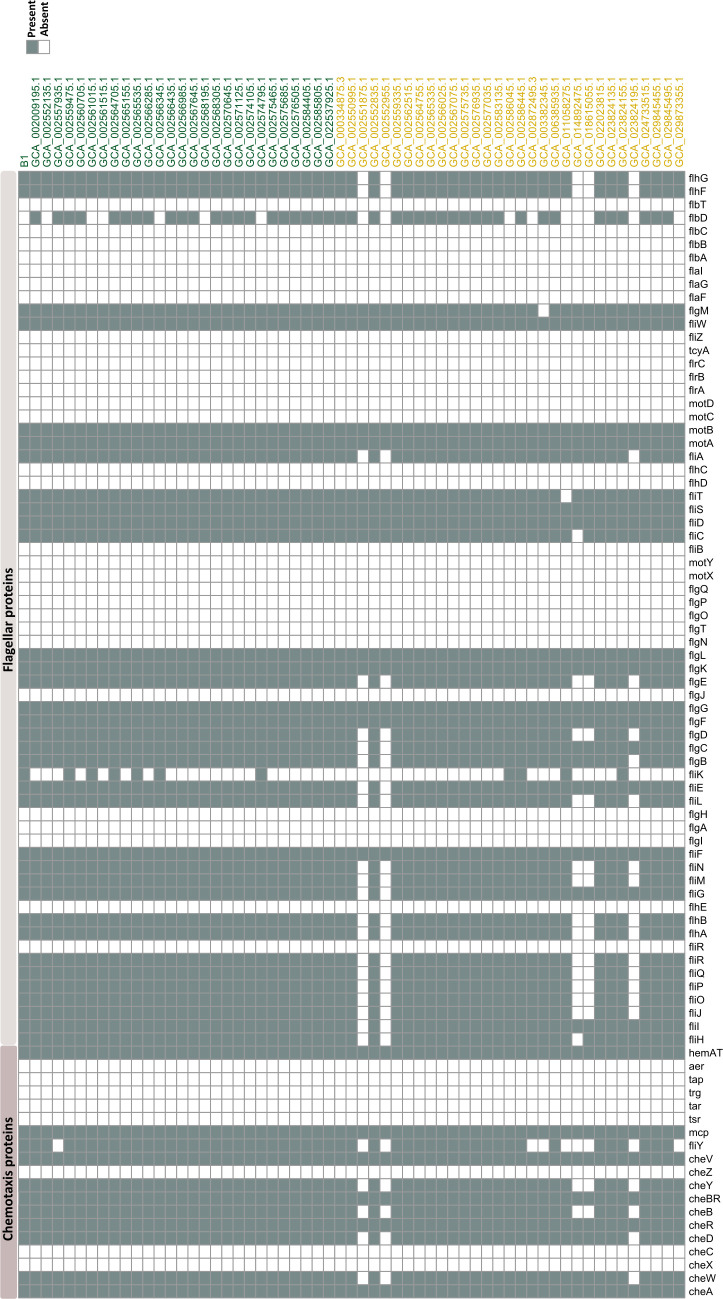
Heatmap showing presence or absence of genes putatively involved in the biosynthesis of chemotaxis and flagellar proteins in plant (green) and soil (brown) strains. Heatmap was generated using R package pheatmap v.1.0.12. Table S5 contains a description of the genes.

Genes encoding chemotaxis and flagellar proteins were screened for each individual strain (Fig. 7). Genomes of all plant strains displayed genes encoding core proteins of the chemotaxis complex, in addition to *cheD* gene. Additionally, the full set of genes essential for flagellar motility were recognized in all strains derived from plant environments, except strain GCA_02557935.1, which lacked the gene encoding C-ring FliY protein. On the contrary, genes coding for the two component system core proteins CheB and CheY were not detected in the genomes of soil strains CCA_014892475.1, GCA_018615055.1, GCA_002551875.1, GCA_002552955.1, and GCA_023824195.1. Genes *cheW* and *cheD* were not identified in strains GCA_002551875.1, GCA_002552955.1, and GCA_023824195.1. Furthermore, strains GCA_002551875.1, GCA_002552955.1, GCA_014892475.1, GCA_018615055.1, and GCA_023824195.1 lacked genes coding type III export proteins (FlhA, FlhB, Flip, FliQ, FliR, and FliJ), hook (FlgE), and C-ring proteins (FliM, FliN, and FliY). Also, strains GCA_002872495.3, GCA_003382345.1, and GCA_029873355.1 did not possess the *fliY* gene. Strains GCA_002551875.1 and GCA_002552955.1 were deficient in genes encoding flagellar basal-body rod proteins FlgB and FlgC, hook protein FliE, flagellar motor switch protein FliG, and type III secretion proteins (FliI and FliH). Moreover, genes encoding flagellar assembly protein FliH and flagellin FliC were not detected in the genome of strain GCA_014892475.1. Strain GCA_023824195.1 was also lacking the rod protein encoding gene (*flgB*). Remarkably, soil strains (GCA_002551875.1, GCA_002552955.1, GCA_014892475.1, GCA_018615055.1, and GCA_023824195.1) exhibited a close clustering in sPLS-DA (Fig. 5) and were closely grouped in the phylogenetic tree, despite originating from distinct biogeographical locations (Fig. 3). Genomes of all strains, from both habitats, did not reveal genes encoding the two component system proteins (*cheX*, *cheC*, and *cheZ*) or the the MCPs proteins (*tar*, *tap*, and *aar*). Similarly, the flagellar proteins coding genes *fliR*/*flhb*, *flhE*, *flgI*, *flgA*, *flgH*, *flgJ*, *flgN*, *flgT*, *flgO*, *flgP*, *flgQ*, *motX*, *motY*, *fliB, flhD*, *flhC*, *motC*, *motD*, *flrA*, *flrB*, *flrC*, *tcyA*, *fliZ*, *flaF*, *flag*, *flaI*, *flbA*, *flbB*, *flbC*, and *flbT* were also missing in all genomes. Secretory system-related genes were also investigated (Fig. S5). All genomes displayed genes encoding the subunits of the Tat pathway, Sec translocase pathway, genes encoding partial components of type VII secretory system, and sortase encoding gene (*srtD*). Moreover, all genomes harbored lipopolysaccharide assembly protein, catalase, and superoxide dismutase encoding genes. Additionally, genes encoding multidrug ABC transporters, RNA polymerase sigma-28 factor (*sigK*), and transcription elongation factor GreA (*greA*) were present in all genomes. The genetic potential of all strains for plant growth promotion was also assessed (Fig. S6). Genes involved in synthesis of indole acetic acid were recognized in all genomes. Additionally, all strains possessed genes encoding siderophore permeases YfiZ and YfhA, siderophore transport ATP-binding protein YusV, and siderophore-binding lipoprotein YfiY. The majority of genomes harbored genes involved in biosynthesis of alkaline phosphatases (PhoA/B/D/P/R), except strain GCA_002872495.3, which was lacking the gene *phoA*, while strains GCA_002561015 and GCA_002567645.1 were deficient in gene *phoB*. Genes encoding malate synthases (*citZ* and *citA*), malate synthase (*glcB*), citrate/malate transporter (*cimH*), and malate transporters (*yfiS*) were detected in all genomes. Moreover, the number of genes encoding families, including CBM, CE, GH, Gt, and PL, was comparable among the strains from both plant and soil habitats, showing no significant difference between the two habitats (*P* value >0.05) (Fig. S7).

## DISCUSSION

### Potential of *P. megaterium* B1 for adapting to plant environment

*P. megaterium* B1 was isolated from the root tissue of healthy apple plantlets and thus considered endophytic. In this study, we presented the genetic and physiological basis of strain B1 to interact with plants and adapt to an endophytic lifestyle. Chemotaxis genetic machinery of strain B1 comprised *mcp* gene, which encodes methyl-accepting chemotaxis protein MCP, along with conserved two-component system genes *cheA*, *cheW*, *cheY*, *cheR*, and *cheB* (24). Additional chemotaxis genes, *cheD* and *fliY* (homolog of *cheC*), were identified, resembling those in *Bacillus subtilis* (25, 26). Previous research involving mutants in *mcp* and *che*A–*che*R genes has demonstrated impaired colonization of plant roots (27–29). These findings highlight the potential of strain B1 to effectively colonize plant roots, as indicated by its chemotactic genetic configuration. Flagella mediate the movement of bacteria toward the roots (1). Earlier studies involving mutations in flagellar-associated genes have demonstrated the critical role of flagellar motility in root colonization (28, 30). The genome of B1 displayed genetic elements necessary for biosynthesis of flagellum components, including the filament, hook, rod, basal body rings, and stator unit, in addition to type III export proteins (31). Additionally, it exhibited both swimming and swarming motilities in biotests. The combined insights from genomic and physiological analyses highlight the motility potential of strain B1, pointing toward a promising capability for successful root colonization. The adherence of the bacterial cells to the root surface can be also mediated through biofilm formation (1). B1 showed the ability to form biofilms and possessed the *lapA* gene needed for biofilm formation (23). Secretory proteins play also a role in plant-microbe interaction (1, 2). The genome of B1 harbors genes encoding Sec and Tat translocase systems, as well as sortase, which are well known in Gram-positive bacteria (32). Moreover, B1 possesses genetic elements related to the type VII secretion system, whose role in promotion of root colonization by *Bacillus velezensis* SQR9 was reported (33). Endophytes are usually challenged by reactive oxygen species (ROS) produced by plants as a defense strategy (2). Thus, genes coding catalase (*katE*) and superoxide dismutase (*sodA*), which are responsible for scavenging of ROS as in B1 further hint to its potential resistance to the plant defense. Additionally, genes encoding multidrug ABC transporter proteins were identified in the genome of B1, which could confer potential resistance to plant immune compounds (e.g., jasmonic and salicylic acids) produced by plants (29). Gene *sigK* encoding sigma 28-factor regulatory protein was also recognized in the genome of strain B1, which plays a role in regulating chemotaxis and motility (34). Additionally, transcriptional elongation factor GreA coding gene, which is also important for plant microbe interaction (35), was identified in the B1 genome. The genome of B1 harbors putative genes coding CAZyme, which facilitate the breakdown of complex compounds into simpler substances, rendering them more accessible for processing and absorption (36). Genes encoding carbohydrate-active enzymes, including those with a role in degrading plant cell walls, were identified in the genome of B1 (37, 38). These involved three families included in hydrolysis of cellulose, hemicellulose, and pectin, which could play a role in facilitating the penetration of plant cell walls by endophytes for subsequent colonization (1). However, production of cell wall components degrading enzymes is critical as it was reported for both endophytes (38) and phytopathogens (39). Furthermore, an α-amylase encoding gene, involved in the hydrolysis of starch (the most common plant reserve carbohydrate), was also detected (37).

### Potential of *P. megaterium* B1 for plant growth promotion

The genomic analysis of strain B1 identified elements related to the synthesis of organic acids and alkaline phosphatases, mechanisms commonly adopted by phosphate-solubilizing bacteria (40). Thus, highlighting B1’s potential to improve phosphorus availability for plants, as it is often inaccessible due to its scarcity in soils and presence in insoluble forms. Similarly, most zinc in soil exists in insoluble complexes, leading to zinc deficiency of plants, a prevalent micronutrient issue. B1’s ability to solubilize zinc oxide, possibly through the production of siderophores and/or organic acids, suggests its capacity to enhance zinc accessibility for plants (41). Additionally, B1’s potential for the synthesis of IAA could play a vital role in plant growth by influencing processes such as root development and photosynthesis (42). However, the concentration of plant-synthesized auxin determines its growth-stimulating or inhibiting effects (42). Bacterial IAA from a plant growth-promoting bacterium may enhance root development in cases of low plant auxin levels or may hinder it when auxin levels are already high (43). Enhancing plant growth and fitness can also be mediated indirectly through antagonizing phytopathogens (44). Mining the B1 genome displayed a biosynthetic cluster encoding surfactins, which is a characteristic lipopeptide of many *Bacillus* strains. Surfactins have been known for their antimicrobial activity against phytopathogens (45, 46), highlighting the antimicrobial potential of strain B1. Interestingly, surfactins were also reported to play an important role in biofilm formation and colonization of plant roots (47), as well as eliciting plant systemic resistance (46). Production of siderophores by B1 can also indirectly inhibit fungal phytopathogens by limiting their access to iron (48), as siderophores produced by plant growth-promoting bacteria possess a higher affinity for iron than fungal siderophores (49).

### Pan-genome and phylogenetic analyses

Pan-genome analysis of *P. megaterium* strains, recovered from soil and plant habitats, displayed a closed pan genome. The closed nature of the pan genome suggests a restricted gene pool, wherein the introduction of a new strain does not contribute to an expansion of the gene repertoire (50). In theory, a bacterial species with a closed pan genome is more likely to thrive in stable environments, such as human or animal tissues, resulting in increased colonization success, in contrast to free-living microorganisms which exhibit higher gene variability to better adapt to diverse environmental conditions (51). Certain host-associated bacteria were indeed documented to have a closed pan genome (52–54). Earlier research demonstrated that obligate intracellular organisms exhibit genomes of smaller size in comparison to their closely related free-living counterparts (55–57). Interestingly, in our case, strains originating from plant and soil environments exhibited no significant differences regarding their genome sizes. While the closed pan genome of selected *P. megaterium* strains hints at a potential for host association, the comparable genome sizes in both plant and soil groups imply a facultative association, suggesting an adaptive lifestyle to both plant and soil environments. To investigate the phylogenetic relationship among plant and soil strains, including our strain B1, and whether the source of isolation or the biogeographical location influences the clustering pattern, a maximum likelihood tree was constructed. Though B1 showed a closer clustering to three plant strains, there was no distinctive clustering pattern based on the habitat or the biogeographical location, which agreed with previous studies involving strains of *Clostridium* (58) and *Methanomassiliicoccales* (59) that reported strains from different habitats to be dispersed in multiple clades.

### Functional comparative genomic analysis

We conducted a functional comparative analysis including plant- and soil-derived strains to investigate the discriminative functional features of the two groups. We performed an sPLS-DA analysis based on Pfam functionally annotated genes, where sPLS-DA plot discriminated the two groups, and the component loading plots identified the most important variables accounting for this variation on both components. However, functional enrichment analysis showed no significant enrichment of Pfams in isolates from one habitat compared to the others. Levy et al. (7) conducted a comparative genomic study encompassing 3,837 genomes from nine taxa, including *Bacillales* (7). Each taxonomic group included strains from various habitats, such as plants (including plant and rhizosphere), roots (encompassing rhizoplane and internal root tissues), soil, and non-plant-associated environments (humans, non-human animals, air, sediments, and aquatic settings). Our analysis focused on Pfam domains significantly associated with plants/roots and soil *Bacillales*. However, we did not observe a significant association of these domains with either plant or soil *P. megaterium* strains. Furthermore, our study identified comparable levels of Pfam LacI transcriptional factor domains (PF00356 and PF13377) in both plant and soil genomes, which were significantly associated with plant-derived genomes in their study. These domains play a crucial role in regulating the expression of genes involved in carbohydrate utilization (60). Additionally, while Levy et al. reported an enrichment of domain PF00248 in plant-associated genomes involved in detoxifying plant-reactive carbonyls (61), our study did not observe significant enrichment of this domain in any specific habitat. In their study, Bünger et al. (8) unveiled that the most significantly enriched features in strains of *Verrucomicrobia*, *Acidobacteria*, *Gemmatimonadetes*, and *Proteobacteria*, originating from the endosphere as opposed to the soil, were associated with flagellar motility. While these features did not display notable enrichment between soil and plant strains, our current examination of potential plant-microbe interaction traits in each individual strain unveiled deficiencies in critical chemotaxis and flagellar genes across five soil strains. Notably, these strains exhibited a close grouping in both the phylogenetic tree and sPLS-DA plot, indicating their relatedness on both phylogenetic and functional levels. Investigating the plant growth-promoting potential of *P. megaterium* strains from both habitats uncovered a broad presence of genetic elements involved in the production of indole-3-acetic acid, biosynthesis of siderophores, and solubilization of phosphate and zinc. This observation is unsurprising, considering that these traits are commonly associated with *P. megaterium* strains recovered from both plant and rhizosphere environments (18–20, 62). Thus, our findings suggest a common set of genetic factors driving the adaptation to plant niches and promoting plant growth in genomes of *P. megaterium* isolates derived from both habitats, plant and soil. This could also imply a conservation of such genetic traits providing strains a certain flexibility to live in bulk soil or at the root soil interface or even to become facultative endophytes, spending parts of their life cycle in the root interior (63). Nevertheless, it is important to consider certain factors when making these conclusions. Firstly, the spore-forming nature of *P. megaterium* strains identified as isolated from soil may primarily consist of dormant spores originating from endophytic strains, awaiting a suitable host for colonization, or vice versa. The second concern lies in the lack of precise specifications regarding the isolation source in the metadata of publicly available genome databases. For instance, when strains are noted as isolated from roots, it remains unclear whether it refers to the root surface (rhizoplane) or the internal tissues. Similarly, for soil strains, the metadata do not provide clear distinctions, leaving ambiguity regarding whether they were isolated from the rhizosphere, bulk soil, or unplanted soil. While comparative genomics is highly advantageous, employing additional methodologies, particularly for closely related strains with similar genetic machinery, is essential to uncover the competence of plant strains compared to their soil counterparts in terms of interaction with plants and colonization. This was emphasized in the investigation conducted by Yi et al. (34), revealing varying levels of competence in closely related green fluorescent protein (GFP) labeled strains of *Bacillus mycoides* recovered from both plant and soil, where the endophytic strain demonstrated higher competence in colonizing plant roots. This observation was complemented by transcriptomic analysis, which revealed distinct expression responses when the strains were exposed to the root exudates of the same plant.

### Conclusion and outlook

In conclusion, our study highlights the physiological and genomic potentials of *P. megaterium* B1 to adapt to the plant niche and promote plant growth. Comparative genomic analysis of strains recovered from plant and soil origins suggests a shared genetic machinery for putative endophytism. This is underscored by their closed pan genome and comparable genome size, suggesting that these strains may function as facultative endophytes capable of transitioning between free-living and host-associated lifestyles. The conservation of plant growth-promoting traits across all strains is advantageous for their broad applicability as bioinoculants in diverse environments. However, the expression of these genomic traits in different environmental conditions should be investigated thoroughly. Additionally, validating the plant growth-promoting capacity of *P. megaterium* B1 for future agricultural applications necessitates further *in planta* investigations. This involves assessing its colonization potential, applying qualitative and quantitative detection techniques such as GFP labeling, fluorescent *in situ* hybridization, and quantitative PCR.

## MATERIALS AND METHODS

### Genome sequencing, assembly, and annotation

The genome presented here is an updated version of the genome published by Mahmoud et al. (22). To obtain a genome of even higher quality, DNA was extracted as described by Mahmoud et al. (22) and the genome was re-sequenced using both PacBio Sequel *IIe* (Pacific Biosciences, Menlo Park, CA, USA) and Illumina MiSeq instruments (Illumina, San Diego, CA, USA). SMRTbell template library was prepared according to the instructions from Pacific Biosciences following the Procedure & Checklist – Preparing Multiplexed Microbial Libraries Using SMRTbell Express Template Prep Kit v.2.0. Briefly, for preparation of 10-kb libraries, 1-µg genomic DNA was sheared using the Megaruptor v.3 (Diagenode, Denville, NJ, USA) according to the manufacturer’s instructions. DNA was end-repaired and ligated to barcoded adapters applying components from the SMRTbell Express Template Prep Kit 2.0 (Pacific Biosciences). Samples were pooled according to the calculations provided by the Microbial Multiplexing Calculator. Conditions for annealing of sequencing primers and binding of polymerase to purified SMRTbell template were assessed with the Calculator in SMRTlink (Pacific Biosciences). Libraries were sequenced using one 15-h movie per SMRT cell. In total, two SMRT cells were run. For Illumina sequencing, a metagenomic library was prepared following the protocol “Metagenomic Library Preparation Protocol using NEBNext Ultra II FS DNA Library Prep Kit (enzymatic shearing),” for high DNA input, using the NEBNext Ultra II FS DNA Library Prep Kit (E7805, E6177) (New England Biolabs GmbH, Frankfurt am Main, Germany). For adaptor ligation and enrichment of adaptor ligated DNA, the NEBNext Multiplex Oligos for Illumina (Dual Index Primers, NEB # E7600; New England Biolabs) was used. The adaptor was diluted 1:10 in sterile diethylpyrocarbonate (DEPC) treated water. Metagenomic libraries were purified using MagSi NGSprep Plus beads (Steinbrenner, Wiesenbach, Germany). For size selection of adaptor-ligated DNA, beads were used in the ratios 0.2*X* and 0.1*X* for 1st and 2nd bead selection, respectively. PCR enrichment of the adaptor-ligated DNA was done using 10 cycles. The quality and quantity of the libraries were checked using the Fragment Analyzer (Agilent Technologies, Santa Clara, CA, USA) using the NGS Fragment Kit (1–6,000 bp) (Agilent Technologies). Finally, the library was diluted to 4 nM. For sequencing, the MiSeq Reagent kit v.3 (600 cycles) (Illumina) was used for paired-end sequencing on the MiSeq instrument (Illumina).

SAMtools v.1.12 was used to obtain PacBio reads in FASTQ format (64). The quality of the PacBio reads was confirmed using LongQC v.1.2.0c (65), and no error correction was performed. Illumina read quality was checked with FastQC (http://www.bioinformatics.babraham.ac.uk/projects/fastqc) (implemented in Galaxy web server, https://usegalaxy.org/). Trimming of adapters, filtering of low-quality reads (<20), and cropping of low-quality sequences (101 bp was trimmed from each direction) were done using trimmomatic v.0.38.1 (66) (implemented in Galaxy web server). PacBio reads were *de novo* assembled using Flye v.2.8.1 (https://github.com/fenderglass/Flye) applying the command “flye--pacbio-raw,” which included circularization of the contigs. The produced assembly was further polished using the trimmed illumine reads applying Pilon v.1.20.1, with three iterations. Plasmids were identified using RFPlasmid v.0.0.18 (67). Completeness and contamination percentages were calculated using CheckM v1.0.18 (68), while QUAST v4.4 was used to check the assembly quality (69). The taxonomic position of strain B1 was checked using digital DNA-DNA hybridization provided by TYGS (70), were it displayed 73.8% similarity with type strain *Priestia megaterium* American Type Culture Collection 14581 (Table S6). The genome was annotated using Prokka v.1.14.6 (71). Functional classification of B1 annotated genes was performed based on COG assignment using EGGNOG-MAPPER v.2.1.11 (72–74), Kyoto Encyclopedia of Genes and Genomes (KEGG) (75) and Pfam database [using InterProScan v.5.65–97.0 (76)]. Putative genes encoding carbohydrate-active enzymes were also analyzed using automated Carbohydrate-Active Enzyme Annotation Server (dbCAN3) (https://bcb.unl.edu/dbCAN2/index.php), which included dbCAN CAZymes domain (by HMMER search), CAZyme subfamilies (by HMMER), and CAZy databases (by DIAMOND search) (77). Prediction of biosynthetic gene clusters was performed using antiSMASH v.7.0.1 (78).

### Physiological potential of *P. megaterium* B1 for adapting to plant environment and enhancing plant growth

To assess **biofilm formation**, 10 µL of overnight culture (OD_600_ = 0.1, ~6 × 10^6^ CFU/mL) was added to 140 µL of nutrient broth medium in a 96-well plate and incubated statically at 30°C. Biofilm formation was quantified after 48 h according to Weng et al. (79) with modifications (79). The medium was drawn off carefully followed by washing with 150-µL sterile distilled water and fixed with 150 µL of 99% (vol/vol) methanol (Fisher Scientific UK Ltd, Leicester, UK) then air-dried. The dried biofilms were stained with 150 mL of crystal violet (CV) solution (Sigma-Aldrich Chemie, Steinheim, Germany) (diluted 1:10) for 30 min. Excess CV was then removed followed by washing using 150 µL of sterile distilled water. The CV bound to the cells was dissolved in 150 µL of 33% (vol/vol) glacial acetic acid (Merk KGaA, Darmstadt, Germany), then optical density was measured using Tecan SparkControl Magellan v.2.2 at 570 nm. Forty replicates were used and glacial acetic acid was used as blank.

**Swimming and swarming motility** tests were performed according to Lucero et al. (80) using nutrient broth (Roth, Karlsruhe, Germany) medium supplemented with 0.3% and 0.5% agar (Becton, Dickinson and Company, Maryland, USA), respectively (80). The medium was poured and allowed to solidify for 30 min in a laminar flow. Three microliters of overnight culture was inoculated in the center of the plate and allowed to dry for 15 min, followed by incubation at 30°C up to 48 h. Five replicates were used.

**Production of indole acetic acid** by B1 was tested following the protocol described by Bric et al. (81) with modifications (81). Ten microliters of overnight culture of B1 was inoculated in 5 mL of Luria-Bertani broth (Roth) supplemented with 5-mM tryptophan (Sigma-Aldrich Chemie) followed by incubation at 30°C for 24 h with shaking (180 rpm). Cells were centrifuged for 10 min at 3,273 × *g* (Allegra X-12, Germany). One milliliter of the cell free supernatant was mixed with 2 mL of Salkowski reagent [1.2% FeCl_3_ (Sigma-Aldrich Chemie) in 37% sulfuric acid (Sigma-Aldrich Chemie)] then incubated for 30 min in the dark. The positive result was indicated by the formation of orange-reddish color. Optical density of the developed color was measured at 530 nm. A standard curve was prepared from commercial indole-3-acetic acid (Sigma-Aldrich Chemie) with concentrations ranging from 1.5625 to 50.0 µg/mL. Five replicates were used.

**Siderophore production** was tested according to Pérez-Miranda et al. (82) and Louden et al. (83) with modifications (82, 83). A single colony of overnight culture of B1 was inoculated in the center of a nutrient agar plate and incubated for 24 h at 30°C. Dye solutions [chrome azurol blue S (MP Biomedicals, Illkirch, France), FeCl_3_ (Sigma-Aldrich Chemie), and hexadecyltrimethylammonium bromide (Sigma-Aldrich Chemie)] were prepared and mixed following the method of Louden et al. (83). Piperazin-N,N′-bis-(2-ethanesulfonic acid) (Pipes, Sigma-Aldrich Chemie) was added to distilled H_2_O with 1% agar (Becton, Dickinson and Company), and pH was adjusted to 6.8. After autoclaving separately, the dye solution was slowly mixed with the Pipes-agar mix. Cooled but still liquid overlay agar (10 mL) was poured on plates cultured with B1, then incubated up to 7 days. Siderophore production was detected by changing the color from blue to orange around the bacterial growth. The test was done using five replicates.

**Solubilization of phosphate** was tested on Pikovskayas agar medium (HiMedia, Mumbai, India). Twenty days after streaking a single colony of overnight culture, phosphate solubilization was determined by formation of a halo zone surrounding the bacterial colony, and the phosphate SI was calculated as (colony diameter + halo zone diameter) / colony diameter.

**Solubilization of zinc** was tested on zinc solubilization agar medium containing (g/L): glucose 10.0, (NH_4_)_2_SO_4_ 1.0, KCl 0.2, K_2_HPO_4_ 0.1, MgSO_4_ 0.2, ZnO 1, agar (Becton, Dickinson and Company) 15, and distilled water 1,000 mL, and buffered to pH 7.0 (15). B1 was inoculated from an overnight culture in the center of the agar plate. After 7 days of incubation at 30°C, solubilization of zinc was detected by the clearance surrounding the colony and expressed as zinc SI: (colony diameter + halo zone diameter) / colony diameter.

### Reference genome data set

We downloaded genomes of *P. megaterium* strains from the National Center for Biotechnology Information GenBank, selecting those clearly identified as isolated from plants or soil for our study. The quality of the genomes was assessed based on the completeness and contamination percentages provided by CheckM v.1.2.2 (68), in addition to the assembly level. Only genomes that displayed completeness of ≥96%, contamination of ≤3%, and assembly level (complete, chromosome, or scaffold) were selected for downstream analysis. In total, 27 and 31 high-quality genomes of plant and soil origins, respectively, were used (Table S7). All genomes were annotated using Prokka v.1.14.6 (71). Functional classification of annotated genes was performed based on COG assignment using EGGNOG-MAPPER v.2.1.11 (72–74), KEGG (75), and Pfam database v.36.0 (84) [using InterProScan v.5.65–97.0 (76)]. Putative genes encoding carbohydrate-active enzymes were predicted using automated dbCAN3 (77).

### Pan-genome and phylogenetic analyses

OrthoFinder v.2.5.5 (85, 86) was used to cluster amino acid sequences in a group of orthologous protein (orthogroups) using DIAMOND (87), applying the default parameters. The OrthoFinder output (Orthogroup.GeneCount) was converted to presence-absence matrix and used to partition the pan genome into core genome, shell, and cloud protein families. Openness of the pan genome was estimated using Heap’s law, using the function “heaps” in the package micropan v.2.1 (88). The pan genome is considered open when α < 1, whereas α > 1 indicates a closed pan genome (89). Accumulation curves of pan genome and core genome were constructed following the R script, publicly available at https://github.com/isabelschober/proteinortho_curves, applying 100 iterations.

A maximum likelihood tree was constructed by OrthoFinder based on multiple sequence alignments of single-copy core orthogroups, by specifying the “-M msa” option. The default programs MAFFT (90) and FastTree (91) were used for generating the alignment and inferring the tree, respectively, while STRIDE was used to root the tree (92, 93). The tree was visualized and edited in iTOL v.6.8 (94). The ANI was also calculated using fastANI (95).

### Functional comparative genomic analysis

To identify genetic markers related to adaptation to the plant environment, we conducted a comparative analysis. This involved strains of *P. megaterium* obtained from both plant habitats and soil, alongside our strain *P. megaterium* B1. Genes assigned to different Pfam domains were counted for each individual strain. To detect Pfam domains discriminating between strains of plant and soil origins, sparse Partial Least Squares Discriminant Analysis (sPLS-DA) was performed (96), using R package “mixOmics v.6.24.0 (97),” based on Pfam presence-absence matrix. The loading weights of top 20 Pfam domains on components 1 and 2 were plotted using the function plotLoadings(). The arguments (method = “mean,” contrib = “max”) were specified, where the color of the graph bars represents the group (plant or soil) with the higher mean. Enrichment of Pfam domains in plant strains, compared to soil strains, was tested. A contingency table representing the count of each Pfam domain in each of the two habitats was constructed. Fisher’s exact test [R package “stats v.4.3.1” (98)] was used to identify significantly enriched domains, and *P* values were adjusted for multiple testing using the Benjamini-Hochberg method (α = 0.05). Additionally, we obtained the Pfam domain set commonly associated with plant and root *Bacillales* genomes, as well as the set identified as significant in soil-associated strains by Levy et al. (7). We examined their presence in our data set and assessed whether they exhibited significant enrichment in the two groups when compared to each other.

Also, potential genes involved in chemotaxis, motility, flagella biosynthesis, secretory systems, stress protection, transcription regulation, as well as plant growth promotion traits, including indole-3-acetic acid production, biosynthesis of siderophores, and phosphate solubilization, were screened for each strain. A heatmap was constructed using the package pheatmap v.1.0.12 (http://cran.nexr.com/web/packages/pheatmap/index.html) to ease visual comparison of these genes among strains from different habitats.

Additionally, genes encoding carbohydrate-active enzymes were predicted using dbCAN3 (77) and counted for each strain. Only, genes that were assigned by the three databases dbCAN CAZymes domain (by HMMER search), CAZyme subfamilies (by HMMER), and CAZy databases (by DIAMOND search) were considered. In case a gene is assigned to more than one family, only the one in common of the three databases was taken in consideration. The four families were tested for significant difference between the two habitats using Wilcoxon test, implemented in R package rstatix v.0.7.2 (99).

## Data Availability

The assembly and annotation, as well as the raw reads, are available via BioProject PRJNA700828. The assembled genome’s accession number is GCA_024582855.4. The new PacBio and Illumina raw reads can be found under the accession numbers SRX23571306 and SRX23584000, respectively.

## SUPPLEMENTAL MATERIAL

Supplemental figures spectrum.00422-24-s0001.docx Fig. S1-S7.

Supplemental tables spectrum.00422-24-s0002.xlsx Tables S1-S7.

ASM does not own the copyrights to Supplemental Material that may be linked to, or accessed through, an article. The authors have granted ASM a non-exclusive, world-wide license to publish the Supplemental Material files. Please contact the corresponding author directly for reuse.
