# Supplementary material for: Comparative genomic analysis of strain Priestia megaterium B1 reveals conserved potential for adaptation to endophytism and plant growth promotion
Source: Microbiol Spectr. 2024 Jun 25;12(8):e00422-24. doi: 10.1128/spectrum.00422-24 (PMC11302069; doi:10.1128/spectrum.00422-24)

**Supplementary figures**


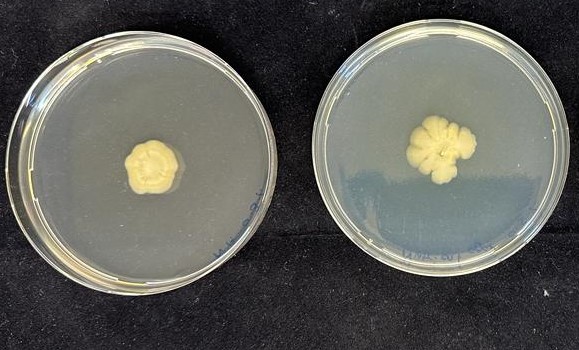


**(B)**

**Figure S1:** Motility test after 48 hours incubation at 30 °C. (A) Swimming motility using 0.3% nutrient agar. (B) Swarming motility using 0.5% nutrient agar.

**(A)**


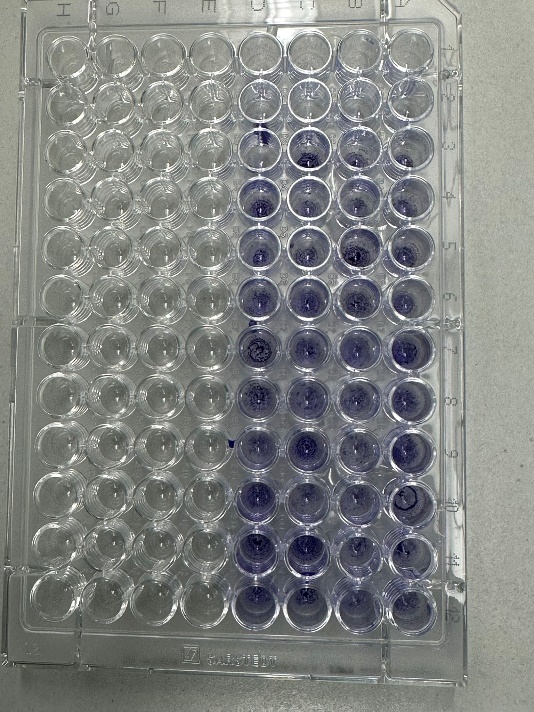


**Figure S2:** Microtitre plate showing the dried crystal-violet stained biofilm, formed by *P. megaterium* B1 after 48 hours incubation at 30 °C. Columns 1 and 2 are the negative controls (only nutrient broth). Columns 3-12 are nutrient broth medium inoculated with *P. megaterium* B1.

**(A)**

**(B)**

**(C)**

**(D)**


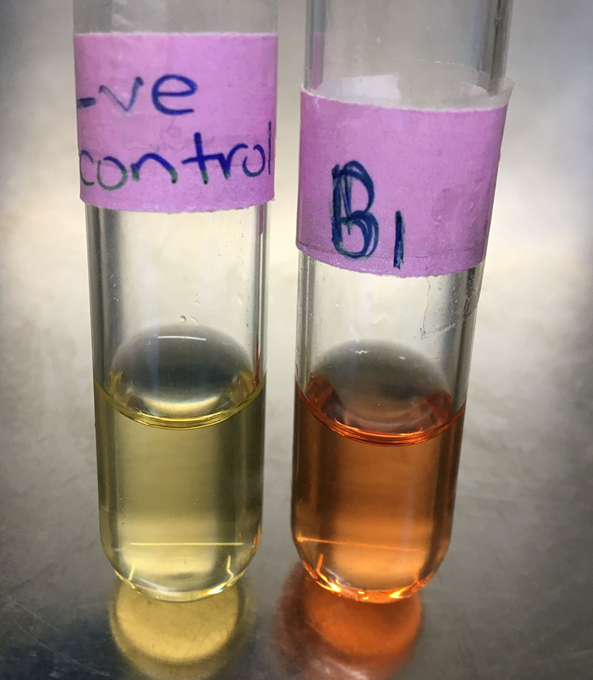

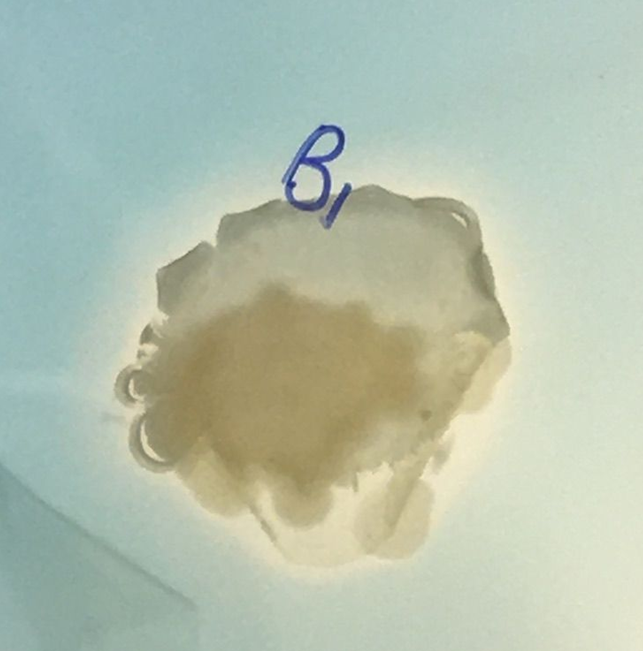


**Figure S3:** Plant growth promoting traits of *Priestia megaterium* B1. **(A)** Indole-3-acetic acid production, **(B)** solubilization of phosphate on Pikovskaya's Agar, **(C)** solubilizationof zinc on zinc oxide supplemented medium, **(D)** production of siderophores on chrome azural S agar .


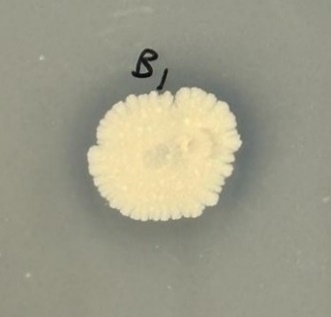

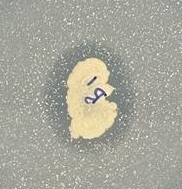

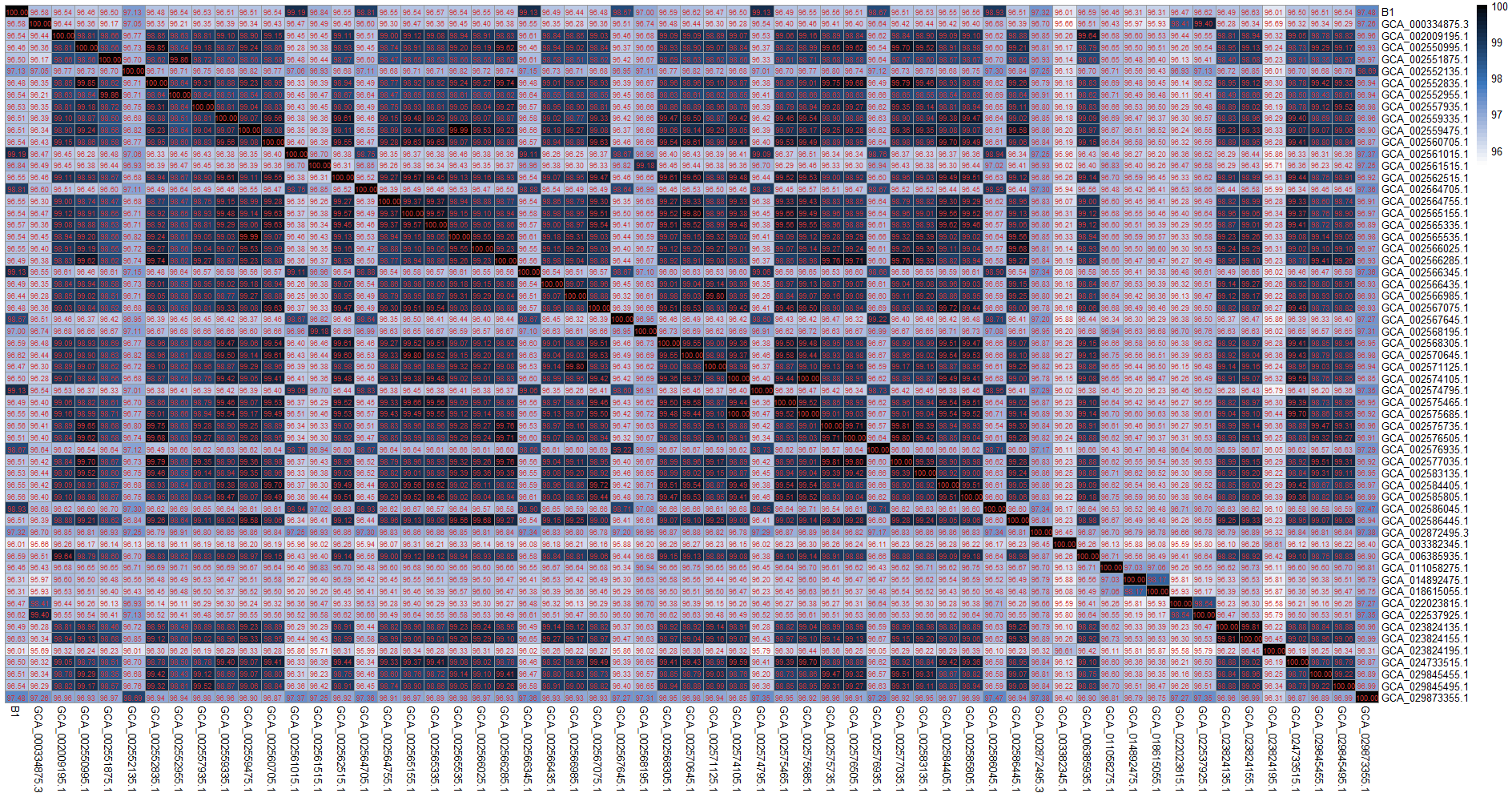


**Figure S4:** Average nucleotide identity of B1 with *P. megaterium* strains of plant and soil origins.

**Figure S5:** Heatmap showing putative genes related to bacterial secretory systems, biofilm formation, detoxification and transcription regulation in plant and soil strains. Heatmap was generated using R package pheatmap v1.0.12. Plant and soil strains are colored green and yellow, respectively.Table S5 contains description of the genes.


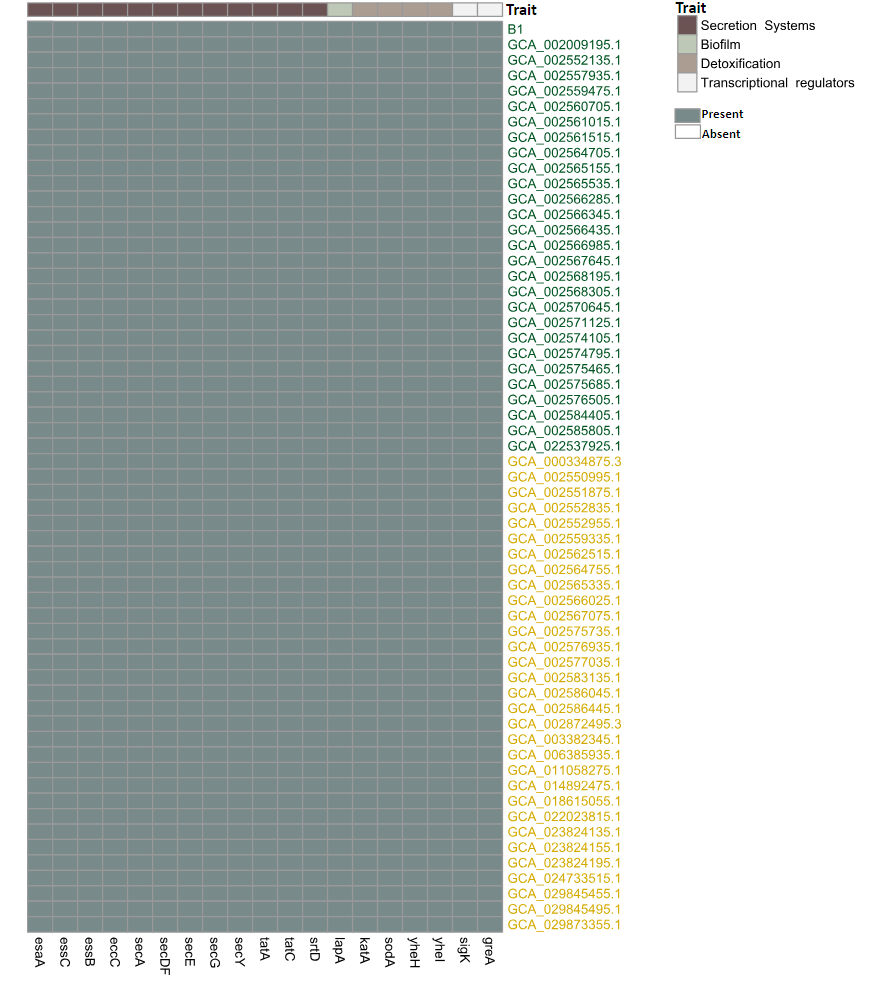

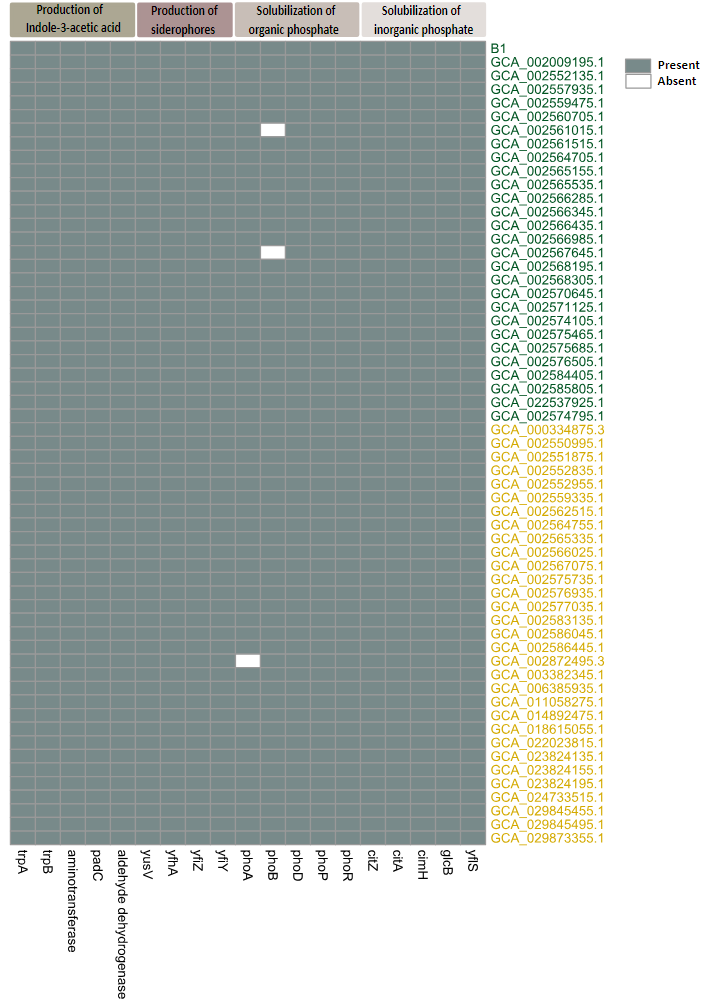


**Figure S6:** Heatmap showing putative genes encoding plant growth promotion traits. Heatmap was generated using R package pheatmap v1.0.12. Plant and soil strains are colored green and yellow, respectively. Table S5 contains description of the genes.

**Figure S7:** Boxplot showing number of genes encoding carbohydrate active enzymes strains recovered from plant and soil habitats. **CBM**: Carbohydrate-Binding Module family, **CE**: Carbohydrate Esterase Family, **GH**: Glycoside Hydrolase family, **GT**: Glycosyltransferase family and **PL**: Polysaccharide Lyase family. P-values were estimated using Wilcoxon test, implemented in R package rstatix v0.7.2


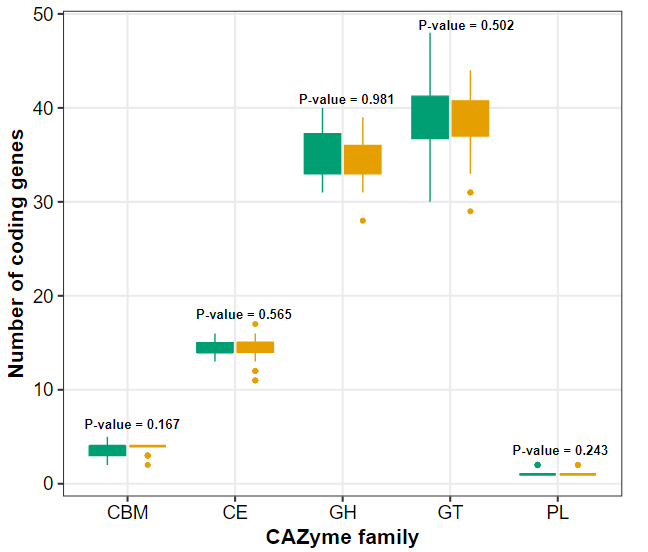

Supplement: Supplemental figures — Fig. S1-S7. [file spectrum.00422-24-s0001.docx]
